# Wide-field time-correlated single photon counting-based fluorescence lifetime imaging microscopy

**DOI:** 10.1016/j.nima.2019.162365

**Published:** 2019-10-21

**Authors:** Klaus Suhling, Liisa M. Hirvonen, Wolfgang Becker, Stefan Smietana, Holger Netz, James Milnes, Thomas Conneely, Alix Le Marois, Ottmar Jagutzki, Fred Festy, Zdeněk Petrášek, Andrew Beeby

**Affiliations:** a Department of Physics, King’s College London, Strand, London WC2R 2LS, UK; b Becker & Hickl GmbH, Nunsdorfer Ring 7-9, 12277 Berlin, Germany; c Photek Ltd, 26 Castleham Rd, St Leonards on Sea TN38 9NS, UK; d Institut für Kernphysik, Max-von-Laue-Str. 1, 60438 Frankfurt, Germany; e Biomaterials, Biomimetics and Biophotonics Research Group, Kings College London Dental Institute at Guys Hospital, Kings Health Partners, Guys Dental Hospital, London Bridge, London SE1 9RT, UK; f Institut für Biotechnologie und Bioprozesstechnik, Technische Universität Graz, Petersgasse, 10-12/I, 8010 Graz, Austria; g Department of Chemistry, University of Durham, Durham DH13LE, UK

## Abstract

Wide-field time-correlated single photon counting detection techniques, where the position and the arrival time of the photons are recorded simultaneously using a camera, have made some advances recently. The technology and instrumentation used for this approach is employed in areas such as nuclear science, mass spectroscopy and positron emission tomography, but here, we discuss some of the wide-field TCSPC methods, for applications in fluorescence microscopy. We describe work by us and others as presented in the Ulitima fast imaging and tracking conference at the Argonne National Laboratory in September 2018, from phosphorescence lifetime imaging (PLIM) microscopy on the microsecond time scale to **fluorescence lifetime imaging (**FLIM) on the nanosecond time scale, and highlight some applications of these techniques.

## 1 Introduction

Optical microscopy is a widely-used tool for non-destructive and minimally invasive observation of living samples. Fluorescence microscopy in particular allows the observation of cell dynamics and function in real time with negligible cytotoxicity. It can also provide high sensitivity, down to the single molecule level, and high specificity. In addition to localising fluorescent labels, the fluorescence can also be used for sensing the immediate environment of the fluorophore, via its spectral properties, its polarisation or fluorescence lifetime [1]. The fluorescence lifetime is often used for this, as it is independent of the concentration of the fluorescence probe, which is difficult to control in cells.

The fluorescence lifetime is the average time a fluorophore remains in the excited state, typically nanoseconds. It can be a function of viscosity, temperature, pH, ion or glucose concentration, refractive index or polarity, and of interaction with other molecules, e.g. due to Förster Resonance Energy Transfer (FRET), a widely used technique to identify protein conformational changes or interactions. Phosphorescence, which originates from the probe’s triplet state, is typically on the microsecond time scale, and is used to sense oxygen concentration, with phosphorescence lifetime imaging (PLIM) being the imaging version of this approach [2], [3]. Fluorescence lifetime imaging (FLIM) and PLIM can measure the lifetimes in every pixel, thus providing image contrast according to the lifetime, which then provides contrast according to viscosity, oxygen or ion concentration or temperature, depending on the type of fluorescent probe employed.

FLIM or PLIM is often carried out with time-correlated single photon counting (TCSPC) and confocal or multiphoton beam scanning. TCSPC is effectively a delayed coincidence method [4], the origin of which lies in particle physics. It can be traced back to 1929, when Bothe and Kohlhörster used two Geiger counters separated by spacers of varying thickness to study coincidences of penetrating charged particles in cosmic rays [5]. This was followed by the first practical electronic coincidence circuit by Bruno Rossi in 1930, which became a precursor of the AND logic gate in electronic circuits [6]. By the addition of a delay, this coincidence method evolved to measure delayed coincidence, and thus provided the means for time-resolved measurements. A method to measure the amplitude of the signal as a function of the delay between pulses by Rossi in 1942, a “time-circuit”, is now known as the time-to-amplitude converter (TAC) [6]. It became a popular method to measure short radioactive decays in the 1950s [7], and in 1961, Bollinger and Thomas generalised the scintillation measurements [8] enabling TCSPC as we now know it, where the arrival time of a single photon is measured relative to an excitation pulse [8]. The accumulation of many single photons then represents the intensity decay of the sample, as long as no photons are lost due to pile-up, and the linearity between intensity and collected photons holds. The first reports that use TCSPC in the measurement of fluorescence decays appear in the early 1970s [9], [10], [11], and TCSPC was soon widely used for time-resolved spectroscopy, and in particular the measurement of fluorescence lifetimes in solutions. Flashlamps used kHz repetition rates [12], but lasers, with picosecond excitation pulses at MHz repetition rates, sped up the measurements significantly and advanced this field enormously [13]. In addition to scintillation and fluorescence measurements, TCSPC is also used for lidar [14] and optical tomography [15].

TCSPC is a sensitive, precise, robust and mature technique to measure photon arrival times after an excitation pulse [4]. Its advantages stem from the digital nature of the technique, based on whether a single photon is detected, or not. It obeys well-defined Poisson statistics, which state that the experimental uncertainty is the square root of the number of counts, and that the signal-to-noise ratio increases with the measurement time, i.e. the number of counts. It also has a large dynamic range in time, from picoseconds to microseconds, and affords an easy visualisation of fluorescence decays. TCSPC has the highest signal-to-noise ratio of the standard time-resolved imaging methods [16], [17], [18], and is precise enough to permit multi-exponential fluorescence decay fitting. By raster scanning the focal spot over the sample the image is created pixel by pixel, using single point detectors to perform TCSPC in each pixel.

While TCSPC is straight-forward to implement with scanning microscopy, there are a number of fluorescence microscopy methods that employ a camera, for example lightsheet microscopy, total internal reflection fluorescence (TIRF), supercritical angle fluorescence and super-resolution fluorescence microscopy methods based on localisation of fluorophores. To harness the advantages of TCSPC for these camera-based fluorescence microscopy methods, and to perform single photon sensitive wide-field FLIM or PLIM with these microscopy methods, a single photon sensitive camera with appropriate time resolution is required [19], [20], [21], [22]. Sensitive detection is favoured, as fluorophores eventually bleach [23]. For microsecond time resolution PLIM, microchannel plate (MCP)-based photon counting image intensifiers with a phosphor screen and a fast camera can be used. For picosecond resolution FLIM, special read-out schemes for image intensifiers, e.g. charge division and propagation time techniques can be employed.

In addition to visible light, single photon detectors based on optoelectronic vacuum devices such as image intensifiers can also detect other types of electromagnetic radiation, including UV photons, X-rays and gamma rays, as well as particles, such as electrons, neutrons and ion fragments. MCPs are used in time-of-flight mass spectroscopy for detecting ion fragments, where molecules in vacuum chamber are ionised and broken up by short laser pulses  [24], [25]. The fragments are accelerated by an electric field towards the MCP, and the position and arrival time contains information about the molecular fragments created. Furthermore, boron-doped MCPs are used for neutron detection and imaging [26], [27], [28], as boron has a high neutron absorption cross section [29]. Timing of the neutron events allows distinction of cold and thermal neutrons [27], and thus enables simultaneous imaging at different neutron energies. This yields different contrast, as absorption cross sections of the material in the object under study are a function of neutron energy. Moreover, in autoradiography, where radioactively labelled samples are imaged, MCPs can be used for sensitive detection of weak β-emitters such as ^3^H, ^14^C, and ^35^S [30]. We note that for these approaches, once a particle has been converted into an electron in the MCP, the detection process is the same as for photons. Thus, development of wide-field detectors for FLIM and PLIM may also benefit these applications.

## 2 Wide-field time-correlated single photon counting imaging techniques and applications

Photon counting imaging, where the image is assembled from individual photons, effectively allows read-out noise free imaging. The approach goes back to the 1980s [33] and was, for example, used on the Faint Object Camera on the Hubble Space Telescope [34]. Note that the prime consideration at the time was sensitivity, not photon arrival timing. Modern MCP-based image intensifiers operated at saturated gain can perform photon counting imaging, and can either be equipped with a phosphor screen output, to be viewed with a camera, or with a position-sensitive anode.

**Fig. 1:**
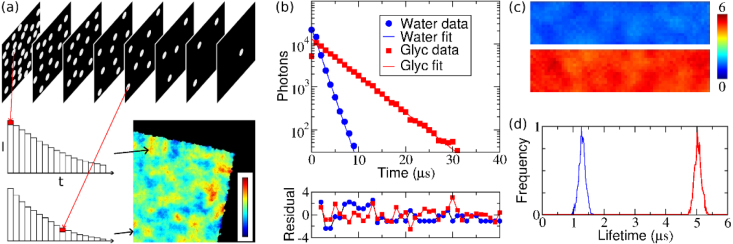
(a) Principle of wide-field TCSPC data acquisition with an MCP intensifier and a CMOS camera. After each excitation pulse, a sequence of frames is acquired. Software searches the frames for position of the photons and their arrival time (i.e. their frame number). These are used to build a histogram of arrival times for each pixel. A lifetime image is obtained by fitting an appropriate exponential decay function to the arrival time histogram in each pixel, and encoding the lifetime in a pseudocolour scale [31], [32]. (b–d) Wide-field TCSPC decays of ruthenium compound Ru(dpp) in water and glycerol solutions, obtained using the principle in (a). (b) Single exponential fits to the data sets with all pixels binned yield lifetimes of 1.35 and 505μs for the water and the glycerol sample, respectively. (c) Lifetime maps of Ru(dpp) in water (top) and glycerol (bottom). (d) Histograms of individual pixel lifetimes in (c). The acquisition time was less than 1 s. (For interpretation of the references to colour in this figure legend, the reader is referred to the web version of this article.)

Adapted with permission from [31].

### 2.1 Image intensifiers with a phosphor screen and camera readout for microsecond decay measurements

#### 2.1.1 Photon arrival time obtained directly from the camera frame rate

Recent developments in imaging sensor technology have allowed complementary metal oxide semiconductor (CMOS) cameras to reach MHz frame rates [31]. These cameras can be used in combination with a photon counting image intensifier for TCSPC [32]. After each excitation pulse, a sequence of frames is acquired during the decay time of the probe, and this process is repeated until enough photons are collected so that a decay histogram is obtained for each pixel of the image, as shown in Fig. 1. The time resolution of this approach is limited by the camera frame rate to the microsecond time scale. Although there is a trade-off between a high frame rate and the number of pixels that can be imaged, this technique enables the collection of up to hundreds of photons per frame, and even several photons after one excitation cycle per pixel as long as they arrive in different frames [32]. Latest developments of sensors that detect a signal above a certain threshold, e.g. timepix [35], [36] or pimms [37] cameras, can also be used for this purpose [38]. The details of the photon event intensity and area covered (number of camera pixels) is of no importance, only the fact where and when a photon event occurs, and therefore such an approach is eminently feasible.

**Fig. 2:**
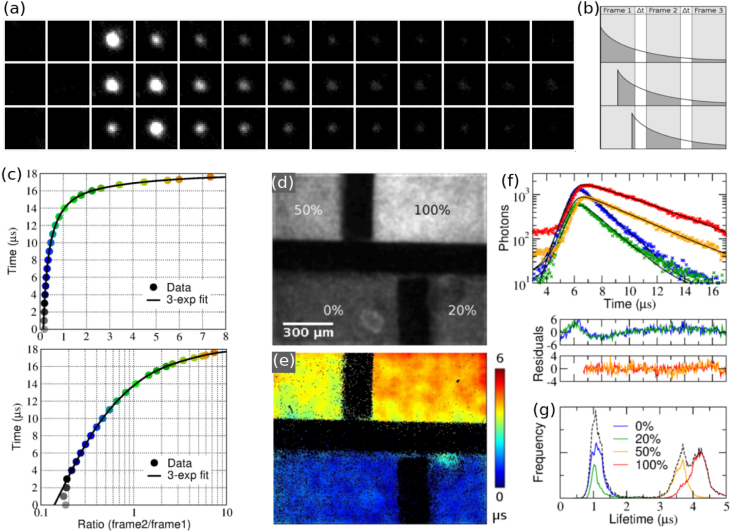
Exploiting the phosphor decay for photon arrival timing within the frame exposure time. (a) Enlarged areas of single photons events arriving at the beginning (top row), in the middle (middle row) and towards the end (bottom row) of the frame exposure time of the 3${}^{rd}$ frame, and (b) a schematic representation of the scenarios in (a). (c) Experimental calibration plot in linear (top) and semi-logarithmic (bottom) scale. (d–g) Lifetime measurement of ruthenium-based luminescent probe Ru(dpp) using the method shown in (a–c): wide-field TCSPC intensity (d) and lifetime (e) images of four Ru(dpp) ＋ water/glycerol solutions, labelled with glycerol % in (d). (f) Decays, fits (black lines) and residuals for the four different areas in (e). (g) Histograms of the individual pixel lifetimes of the different solutions in (e). The dashed line shows histogram of the whole image. The data set colours in (f) correspond to colours in (g).(For interpretation of the references to colour in this figure legend, the reader is referred to the web version of this article.)

Adapted from [39].

#### 2.1.2 Photon arrival time obtained from imaging the phosphor decay

**Fig. 3:**
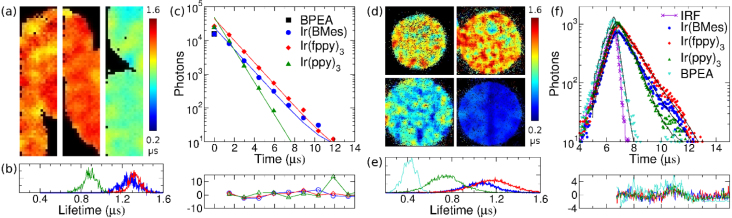
(a) Lifetime images of beads with iridium complexes Ir(ppy)_3_, Ir(BMes) and Ir(fppy)_3_ (from left to right, respectively), imaged with direct TCSPC measurement with 675,000 Hz camera frame rate. (b) Histograms of the individual pixel lifetimes in (a). (c) Decays of images in (a) with all pixels binned, mono-exponential fits to the data (lines) and residuals. (d) Lifetime images of beads with Ir(ppy) (top left), Ir(BMes) (top right), Ir(fppy) (bottom left) complexes and BPEA (bottom right), imaged with phosphor decay method and 54 kHz camera frame rate. (e) Histograms of the individual pixel lifetimes in (c). (f) Decays of images in (c) with all pixels binned (data points), mono-exponential fits to the data (lines) and residuals.(For interpretation of the references to colour in this figure legend, the reader is referred to the web version of this article.)

The phosphor decay of the intensifier output screen can be exploited to find the photon arrival time within the frame exposure time [40]. This effect, an afterglow, is usually undesired [41], but can be put to good use. Matching the camera frame rate to the phosphor decay such that the photon events can be seen in several consecutive frames allows the photon arrival time within the camera exposure time to be found from the relative brightness of the photon event in successive frames, as illustrated in Fig. 2. This approach is similar to dual exposure techniques for velocity map imaging in mass spectroscopy [42], [43]. The measurement of the photon arrival time from the phosphor decay can improve the time resolution beyond the inverse frame rate of the camera, and the lower frame rate increases the number of recorded pixels, thus allowing bigger field of view [39], [44]. Phosphorescent sample decays as short as 500 ns have been measured with a P20 phosphor and 300 kHz frame rate [45], as shown in Fig. 3, but a combination of a faster phosphor and a faster frame rate, or special cameras such as timepix [35], [36] or pimms [37] cameras, could allow the measurement of even faster sample decays.

Camera-based wide-field TCSPC is especially well suited for sensitive measurements of phosphorescence lifetimes in the micro- and millisecond time region, i.e. PLIM [2], [3]. Camera-based methods enable the collection of hundreds of photons per excitation cycle, shortening the data acquisition time with long lifetime probes compared to single point scanning measurements. Lifetimes around 1μs have been measured with several transition metal probes using these techniques, including a ruthenium-based oxygen sensor in living cells, with total image acquisition times of just a few seconds [31], [39]. In fact, the best use of this approach is probably for microsecond lifetime PLIM measurements due to the limited overall count rate of the image intensifier. It enables the collection of many photons per excitation pulse with low excitation power and without lengthy scanning.

### 2.2 Image intensifiers with an electronic anode readout for nanosecond decay measurements

For nanosecond fluorescence lifetime measurements, tens or hundreds of picosecond precision for photon arrival timing is essential. While the MCPs themselves are capable of timing the photon arrival with a precision of a few tens of picoseconds [4], they are not capable of recording the photon’s position without special read-outs. Different read-out architectures have been developed, including quadrant, wedge-and-strip, cross-strip and delay line anodes [19], [20], where the position of the electron cloud is determined via a charge division approach, or via the propagation time along a delay line [46]. Although some of the read-out schemes can accommodate multiple photon events after one excitation cycle, i.e. the hexanode [47] or cross-strip read-outs [19], the count rate is typically limited by the position readout electronics to a few tens or hundreds of kHz, rather than the need to avoid overlapping events. However, the advantage here is that sub-microwatt excitation powers are sufficient to generate photons, and allow continuous observation of living samples over days. Wide-field data collection allows the tracking of individual molecules or particle trajectories, e.g. single quantum dot tracking and FLIM has been demonstrated with a MCP and delay line anode detector [48]. Quadrant anodes [49], [50] have been applied to FLIM for the study of protein–protein interaction by FRET [51], [52], [53] and photosynthesis [54], [55].

Wide-field TCSPC is especially useful for microscopy methods where the whole field of view is illuminated with a technique that provides depth discrimination. One of these techniques is TIRF microscopy, where the sample is excited by an evanescent wave only near (up to 100 nm) the coverslip. Total internal reflection (TIR)-FLIM has been demonstrated with quadrant anodes [56] and single photon avalanche diode (SPAD) detectors [57].

**Fig. 4:**
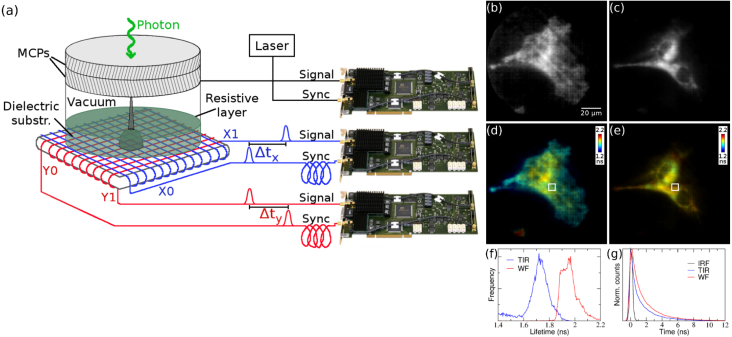
(a) In a delay line anode, the position of the photon event is obtained from the signal propagation time difference to the ends of the delay line. Multiple photon events can be discarded by a run-time check, or timed with a third delay line (hexanode). The timing can be performed with three standard TCSPC boards, one for *x*, one for *y* and the third for time, *t*. (b–e) TCSPC images of fixed HeLa cells, stained with membrane dye laurdan, acquired with a delay line anode detector. The measured intensity shows the cell membrane only under TIRF illumination (b), and the fluorescence lifetime of laurdan is shorter (d), while the whole cell is visible under wide-field illumination (c) and the laurdan lifetime is longer (e). (f) Histograms of the individual pixel lifetimes in (c, d). (g) Fluorescence decays in the area indicated by a white rectangle are shown in (c, d). The instrument response function, measured with reflection, has a FWHM of 344 ps . (For interpretation of the references to colour in this figure legend, the reader is referred to the web version of this article.)

Reprinted from [59], with the permission of AIP Publishing.

We have used a delay line anode detector (Photek) where the delay line is capacitively coupled to a resistive anode inside the tube and using an image charge technique [20], [58], to perform TIR-FLIM [59]. Fig. 4 shows wide-field TCSPC FLIM images of fixed HeLa cells acquired with this delay line anode detector, read out by conventional TCSPC timing boards (Becker & Hickl) [60]. The sample was excited with a Horiba DeltaDiode picosecond laser (375 nm) at 10 MHz, and the photon count rate was around 80 kHz, with an acquisition time of 100 s. The measured intensity shows the cell membrane stained with membrane dye laurdan only under TIRF illumination (Fig. 4b), while the whole cell is visible under wide-field illumination (Fig. 4c). The laurdan fluorescence lifetime is also shortened under TIRF illumination (Fig. 4d) compared to wide-field illumination (Fig. 4f); one contributory factor here is the proximity of the high refractive index glass coverslip which consequently shortens the fluorescence lifetime [61]. Fig. 4g shows the measured instrument response function (IRF), and the measured time decays in a small area in Fig. 4c, d. The IRF full width at half maximum (FWHM) is 344 ps.

The low excitation power used in wide-field TCSPC can help to minimise photodamage in living cells which is especially beneficial for observing dynamics in living cells over long periods.

### 2.3 Electron-bombarded sensors

Single photon detection is also possible with electron-bombarded (EB) sensors, where the photoelectrons from the photocathode are accelerated directly into a CCD or CMOS sensor [62], [63], [64], [65], [66]. Unlike MCPs where the statistical electron multiplication process creates a broad pulse height distribution, in EB sensors the photon event brightness depends on the gain voltage. Thus, by sweeping the gain voltage during the exposure time, it could be possible to obtain photon arrival time information from the photon event brightness, akin to a 2-dimensional streak camera [62]. The concept has been proposed and simulated, but not implemented; gain sweeping has not been possible with commercially available devices.

### 2.4 Single photon avalanche diode (SPAD) arrays

Another option to obtain a position-sensitive single photon detector is to build an array of single photon sensitive point detectors [67], [68], [69]. A single photon avalanche diode (SPAD, reverse biased above the diode breakdown voltage, Geiger mode) is a small all-solid state photon detector with a diameter of a few microns, and is capable of single photon detection with picosecond time resolution [70]. Single SPADs were first used for fast timing applications in the 1980s, and the implementation of SPADs in CMOS technology in 2003 enabled the development of SPAD arrays [70]. Unlike image intensifiers or EB sensors, SPADs do not require a high voltage or a vacuum, they are not damaged by high light levels, and they can be manufactured in arrays, 256×256 pixel [71] and 240×320 pixel SPAD arrays [72] have been reported.

SPAD arrays are a relatively new development in wide-field TCSPC. Initially, gated SPAD arrays for fluorescence lifetime were implemented [73], but now each photon can be timed individually, and in all pixels in parallel [70]. The big advantage of these developments in SPAD array detector technology is that it allows independent TCSPC in each pixel of a SPAD array simultaneously, e.g. in the 32 × 32 pixel megaframe chip, with a TDC in each pixel, with 55 ps resolution [74], [75]. They simultaneously deliver single photon sensitivity, tens of thousands of pixels spatial resolution and picosecond timing resolution [70], [76], [77]. The outstanding capability of enormous global count rates well into the gigahertz region [78], which would allow the observation of fast cellular dynamics, is a big advantage of these devices.

The design of detectors and timing electronics on a single substrate inevitably provides compactness and large numbers of channels but compromises fill-factor and SPAD performance (jitter, photon detection efficiency, after pulsing and dark count). Nevertheless, SPAD array technology offers a huge advantage over existing FLIM detector technology. Most SPAD array detectors currently have a small fill factor (<10%), because the majority of the area of each pixel is occupied by electronic circuits to perform the timing, with only a small light-sensitive area dedicated to the detection of photons. Promising current developments in 3D stacking of integrated circuits [79] will ensure a fill factor >80%, a better time resolution and reduced jitter. Moreover, logic integration will scale up enormously enabling placement of field programmable gate arrays-like structures beneath the sensor. The development of 100% fill factor SPAD arrays will not only allow fast fluorescence lifetime measurements via wide-field TCSPC FLIM, the sensitivity and speed of this kind of detector could also benefit other applications, and this field continues to develop at a fast pace [70], [77]. SPAD arrays are also used for positron emission tomography, where the picosecond timing capabilities can pinpoint the localisation of the annihilation event more precisely than without timing, and thus increase the spatial resolution of the technique [80]. In addition, SPAD arrays have been used for detection of Cherenkov radiation in radiation therapy [81]. Range-finding is another application of SPAD arrays [14], for example consumer electronics such as mobile phones have benefitted from TCSPC-based range finding, where sensitivity is not as big an issue as it is with FLIM.^4^

## 3 Conclusions

Fluorescence microscopy allows non-destructive and minimally invasive observation of living samples, and FLIM and PLIM allow the monitoring of the microenvironment of fluorescence and phosphorescence probes. A photon counting approach to FLIM and PLIM is particularly helpful, as it minimises light exposure of the sample. It also is the best method to collect the fluorescence from the sample before the flurophores are irreversibly bleached [23]. Wide-field TCSPC-based methods combine the advantages of single photon sensitivity and precision with wide-field data collection. This is important for implementation of specialised FLIM and PLIM microscopy methods that typically employ cameras, such as TIRF, lightsheet and others.

We have shown that wide-field time-correlated single photon counting based on an image intensifier with a phosphor screen and a fast CMOS camera can be employed for PLIM to map phosphorescent decays on a microsecond time scale. This can either be done with direct imaging of the photon events on the image intensifier’s phosphor screen [31], or by exploiting the invariant phosphor decay of the image intensifier screen for accurate timing of photon arrival well below the camera exposure time [39]. To image nanosecond fluorescence decays, we show that a crossed delay line anode detector read out with conventional TCSPC boards is feasible [59]. This approach retains all the advantages of TCSPC, and extends them to wide-field detection, serving essentially as a single photon sensitive camera with picosecond time resolution. Application of this approach to TIR FLIM using the membrane dye laurdan shows lifetime contrast between the plasma membrane and the interior membranes of the cell, as shown in Fig. 4. Photon counting approaches are particularly useful for fluorescence microscopy methods employing a camera to enable TCSPC-based FLIM, the FLIM method with the highest signal-to-noise ratio. The extremely low illumination intensity, distributed evenly over the field of view, is beneficial especially in life science applications where it allows long-term monitoring of living cells and organisms, while wide-field data collection enables the observation of cell dynamics and single particle tracking. A position-sensitive photon counting detector has recently been used for simultaneous acquisition of both spectral and temporal information of Raman photons from tissue phantoms [82], showing the versatility of these types of detector.

Wide-field TCSPC methods are currently mainly based on MCP-based detectors, a mature technology used especially in astronomy and medical imaging. However, the development of SPAD arrays and their application to FLIM with the prospect of huge photon count rates continues at a rapid pace.

## Footnotes

4 See http://www.st.com/content/st_com/en/about/media-center/press-item.html/stmicroelectronics-proximity-sensor-solves-smartphone-hang-ups.html for information about proximity sensing in mobile phones using photon time-of-flight measurements with SPAD arrays.
